# The effects of denosumab and alendronate on glucocorticoid-induced osteoporosis in patients with glomerular disease: A randomized, controlled trial

**DOI:** 10.1371/journal.pone.0193846

**Published:** 2018-03-15

**Authors:** Ken Iseri, Masayuki Iyoda, Makoto Watanabe, Kei Matsumoto, Daisuke Sanada, Takashi Inoue, Shohei Tachibana, Takanori Shibata

**Affiliations:** 1 Division of Nephrology, Department of Medicine, Showa University School of Medicine, Tokyo, Japan; 2 Nephrology Center, Makita General Hospital, Tokyo, Japan; Universidade Nove de Julho, BRAZIL

## Abstract

**Introduction:**

The clinical utility of denosumab for the treatment of glucocorticoid-induced osteoporosis (GIOP) has yet to be established. This study aimed to compare the effects of denosumab on bone mineral density (BMD) and bone turnover markers to those of alendronate in patients with GIOP.

**Methods:**

A prospective, single-center study of 32 patients (18 men; median age, 66.0 years) with glomerular disease receiving prednisolone (PSL) who were diagnosed as having GIOP and had not received bisphosphonates before was conducted. Participants were randomized to either alendronate (35 mg orally once a week) or denosumab (60 mg subcutaneously once every 6 months), and all subjects received calcitriol. The primary endpoint was the percent change in lumbar spine (LS) BMD at 12 months of treatment.

**Results:**

The demographic and clinical characteristics at baseline were not significantly different between the groups. Denosumab treatment markedly decreased serum levels of t-PINP, BAP, and TRACP-5b at 12 months compared to baseline (-57.4%, p<0.001; -30.9%, p<0.01; -57.7%, p<0.001, respectively). After 12 months of alendronate treatment, serum levels of t-PINP, BAP, and TRACP-5b were also significantly decreased compared to pretreatment (-38.9%, p<0.01; -16.3%, p<0.05; -43.5%, p<0.01, respectively). However, no significant differences in the changes of bone turnover markers were found between the two groups. As for the effects on BMD, denosumab treatment markedly increased LS BMD from 6 months compared to baseline, whereas no significant difference compared to pretreatment was found in the alendronate group during the study period. In the comparison of the two groups, a large increase of LS BMD was found in the denosumab treatment group compared to the alendronate treatment group at 12 months (p<0.05).

**Conclusions:**

In patients with GIOP, denosumab treatment markedly suppressed bone turnover, which led to a significantly greater increase in LS BMD than with alendronate treatment. These results suggest that denosumab is a therapeutic option for the treatment of GIOP.

## Introduction

Glucocorticoids are widely used for the treatment of various inflammatory and autoimmune diseases, but they have various adverse effects on multiple organ systems. In addition, the risk of fractures is increased in patients treated with glucocorticoids. Glucocorticoid-induced osteoporosis (GIOP), characterized by rapid bone loss within the first months after initiation and slower bone resorption thereafter, is the most common form of secondary osteoporosis [1]. It has been reported that the relative risk of fractures was much higher in patients receiving glucocorticoids independent of bone mineral density (BMD) than in those never treated with glucocorticoids, and long-term glucocorticoid therapy caused bone fractures in 30–50% of patients [2,3]. As for bone protection, bisphosphonates are considered standard therapy for GIOP, but concerns with the use of bisphosphonates in children, young men, and premenopausal women, who are often included in the population with glomerulonephritis, still remain [4].

Denosumab is a fully humanized monoclonal IgG2 antibody against the receptor activator of nuclear factor-κB ligand (RANKL), which is a key effector of osteoclast formation, function, and survival [5]. It has been reported that denosumab treatment rapidly decreased bone resorption, increased BMD at the lumbar spine (LS) and total hip, and reduced the risk of new fractures in postmenopausal women with osteoporosis [6]. Denosumab has the unique characteristic of rapid reversibility of its antiresorptive effect after discontinuation, in contrast to bisphosphonates [7]. This might be preferable in young populations, especially premenopausal women with childbearing potential. To date, denosumab has been widely used not only in postmenopausal osteoporosis, but also other types of osteoporosis, such as secondary hyperparathyroidism, but limited information is available regarding the therapeutic potential of denosumab in patients with GIOP. The purpose of the present study was to compare the efficacy and safety of denosumab with those of alendronate in GIOP patients with glomerular disease.

## Patients and methods

### Study design and participants

This was a 12-month, single-center, open-label, randomized, controlled study that recruited 32 patients with glomerular disease who were diagnosed with GIOP according to Japanese Society for Bone and Mineral Research criteria [8], as follows: patients committed or exposed to ≥ 3 months of oral glucocorticoid therapy whose score was ≥ 3 calculated by clinical risk factors, such as prior fragility fracture, age, glucocorticoid dose, and BMD.

Subjects were randomly assigned in a 1:1 ratio to receive denosumab (60 mg) subcutaneously every 6 months or oral alendronate 35 mg/week for 1 year. All patients received at least calcitriol 0.25 μg/day throughout the study. Medications for the treatment of the underlying diseases were continued as usual. Inclusion criteria were: (1) diagnosed with GIOP; and (2) age > 20 years. Exclusion criteria were: (1) patients who had been pretreated with bisphosphonate or denosumab in the preceding 6 months; (2) eGFR < 35 mL/min/1.73 m^2^; (3) intact-PTH (i-PTH) > 300 pg/mL; (4) corrected Ca < 8.4 mg/dL; and (5) cancer patients. The study protocol was approved by the Showa University Ethics Committee and registered with the University Hospital Medical Information Network Clinical Trials Registry (UMIN-CTR) (number UMIN000019574). All patients provided their informed consent to the study following a careful explanation.

### Assessments

Demographic and baseline characteristics were summarized for the full-analysis-set (FAS) population. The primary endpoint was the percentage change from baseline in LS BMD at 12 months. The secondary endpoints were the percentage change in BMD at other sites and relative changes in bone turnover markers from baseline to 12 months. Tartrate-resistant acid phosphatase 5b (TRACP-5b), bone-specific ALP (BAP), and total-type I collagen N-terminal propeptide (t-PINP) were measured as bone turnover markers not affected by the renal insufficiency. BMDs of the LS, femoral neck (FN), and ultra-distal radius (UD) were measured by dual-energy X-ray absorptiometry (DXA; Discovery A, Hologic Inc., Waltham, MA) at baseline, 6 months, and 12 months. The well-experienced technician who measured BMD was blinded. Radiographs of the lateral lumbar and thoracic spine were taken at baseline, 6 months, and 12 months. Morphometric vertebral fractures were assessed according to the semiquantitative method of Genant et al. [9]. Serum levels of albumin (Alb), calcium (Ca), phosphate (P), uric acid (UA), alkaline phosphatase (ALP), triglycerides (TG), total-cholesterol (TC), HDL-cholesterol (HDL-C), LDL-cholesterol (LDL-C), HbA1c, and i-PTH, as well as the estimated glomerular filtration rate (eGFR), were measured with the autoanalyzer in our hospital before treatment. Serum levels of bone metabolic and quality markers (TRACP-5b, BAP, t-PINP, pentosidine, homocysteine, and 1,25(OH)_2_VitD) were measured at baseline, 6 months, and 12 months. These parameters were measured in a commercial biochemistry laboratory (SRL, Inc., Tokyo, Japan).

All subjects were questioned concerning adverse events, and serum chemistry and hematology values were evaluated at each visit; all adverse events were assessed, regardless of the determinations of causality by the investigators.

### Statistical analyses

Demographic data are presented as means ± standard deviation (SD) or medians (range from the 25th to the 75th percentile) for continuous variables unless otherwise noted and as numbers (percentage) for categorical variables. The Shapiro-Wilk test was used to assess the normality of distributions for continuous variables. Comparisons between two groups for normally distributed variables were performed using Student’s *t*-test, and the Wilcoxon rank sum test was used for non-normally distributed variables. Fisher’s exact test was used to compare nominal variables, and the chi-squared test was used to compare underlying diseases between the two groups. The mean changes in BMD and bone turnover markers are presented as means ± standard error of the mean (SEM). Between-group differences from baseline to 6 months or 12 months were examined by Wilcoxon’s rank sum test. Similarly, within-group changes were assessed by Wilcoxon’s signed rank test. According to the previous reports, it was anticipated that the increase of LS BMD at 12 months by denosumab therapy would be 3% more than by alendronate treatment and that the standard deviation of LS BMD would be 2.5% [10,11]. Sample size calculation (two-sided) estimated that a total of 32 patients (16 patients in each arm) were required to detect significant differences with an α error of 5% and a power of 90%. For all tests, the level of significance was set at p<0.05. All statistical analyses were performed by JMP Pro Ver.13 (SAS Institute, Cary, NC).

## Results

### Baseline characteristics and clinical parameters

**Fig 1:**
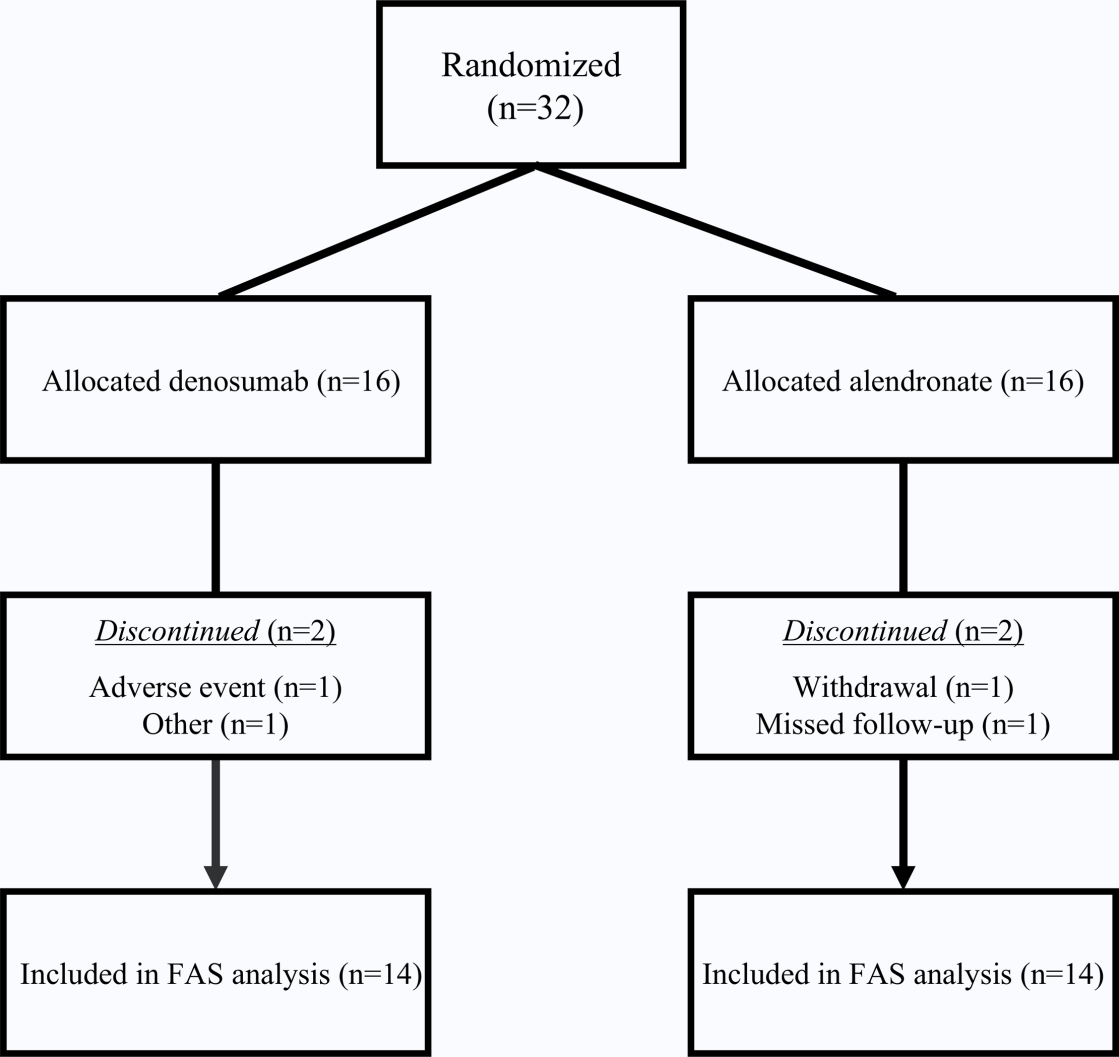
Patient disposition. Abbreviations: FAS, full analysis set.

Thirty-two patients with glomerular disease diagnosed as having GIOP were recruited, and four patients were discontinued (withdrawal [n = 1], missed follow-up [n = 1], adverse event [n = 1], other [n = 1]) (**Fig 1**). Thus, 28 patients (FAS population) were analyzed, and the patients’ baseline characteristics are shown in **Table 1**. For all patients, the median age was 66.0 years, and 43% were female. Postmenopausal women accounted for 9% of the patients. The most common underlying glomerular disease was minimal change nephrotic syndrome (MCNS, 32%), followed by lupus nephritis (25%). Eight patients (29%) had been prescribed glucocorticoids for less than 3 months, and 20 patients (71%) had been treated for at least 3 months. Among patients receiving PSL or vitamin D before the study, the median daily dose of prednisolone (PSL) was 5.0 mg, and the median durations of PSL and vitamin D therapy were 7.3 and 1.5 years, respectively. The GIOP score, previous fractures, and other risk factors for fracture were not significantly different between the denosumab group and the alendronate group.

**Table 1:** Clinical characteristics at baseline.

	Total	Denosumab	Alendronate	*p*
	(N = 28)	(N = 14)	(N = 14)	
**Age—yr**	66.0 (42.5–76.5)	66.5 (39.0–75.8)	65.5 (45.0–78.5)	0.748
**Body mass index (kg/m**^**2**^**)**	22.4 ± 3.8	22.8 ± 3.9	22.0 ± 3.7	0.613
**SMI (skeletal muscle index)**	7.0 ± 1.8	7.3 ± 1.8	6.7 ± 1.8	0.323
**Female/male**	12 / 16	6 / 8	6 / 8	1.000
** Postmenopausal women—no. (%)**	9 (32.1%)	5 (35.7%)	4 (28.6%)	1.000
**Previous drug therapy—no. (%)**				
** Glucocorticoid**	20 (71.4%)	10 (71.4%)	10 (71.4%)	1.000
** Prednisone equivalent daily dose—mg**	5.0 (2.5–8.8)	5.0 (2.4–8.5)	5.0 (2.5–9.3)	0.788
** Duration of therapy—yr**	7.3 (1.9–17.5)	6.9 (2.2–19.0)	9.0 (1.8–19.1)	0.909
** Vitamin D**	18 (64.3%)	9 (64.3%)	9 (64.3%)	1.000
** Eldecalcitol (0.75 μg/day)**	10 (35.7%)	5 (35.7%)	5 (35.7%)	
Alfacalcidol (0.25 μg/day)*	7 (25%)	3 (21.4%)	4 (28.6%)	
** Calcitriol (0.25 μg/day)**	1 (3.5%)	1 (7.0%)	0 (0%)	
** Duration of therapy—yr**	1.5 (0.2–11.9)	2.5 (0.1–10.5)	1.1 (0.6–12.2)	0.930
**GIOP score**	6 (4–10)	6 (4–8)	7.5 (4–12.3)	0.590
**Previous fracture—no. (%)**	5 (17.9%)	2 (14.3%)	3 (21.4%)	0.596
**Current Smoker—no. (%)**	10 (35.7%)	5 (35.7%)	5 (35.7%)	1.000
**Habitual drinking—no. (%)**	2 (7.1%)	2 (14.3%)	0 (0%)	0.482
**Parent Fractured Hip**	1 (3.5%)	0 (0%)	1 (7.1%)	1.000
**Rheumatoid arthritis**	1 (3.5%)	0 (0%)	1 (7.1%)	1.000
**Underlying disease**				0.605
** MCNS**	9	4	5	
** Lupus nephritis**	7	3	4	
** MN**	5	2	3	
** ANCA-GN**	3	3	0	
** FSGS**	2	1	1	
** IgAN**	1	1	0	
** HSPN**	1	0	1	

Data are presented as mean ± SD, median (range from 25th to 75th percentile), or n (%).

Abbreviations: GIOP score, glucocorticoid-induced osteoporosis score; MCNS, minimal change nephrotic syndrome; MN, membranous nephropathy; ANCA-GN, antineutrophil cytoplasmic antibody-associated glomerulonephritis; FSGS, focal segmental glomerulosclerosis; IgAN, immunoglobulin A nephropathy; HSPN, Henoch-Schönlein purpura nephritis

*Alfacalcidol: 1 patient 1.0 μg/day, 6 patients 0.25 μg/day

**Table 2** shows the baseline clinical parameters and BMD. The median eGFR values of the denosumab- and alendronate-treated patients were 61.1 and 49.0 mL/min/1.73 m^2^, respectively. Corrected Ca, ALP, and i-PTH levels were comparable between the two groups. With regard to bone turnover makers, the median levels of TRACP-5b, a bone resorption marker, of the denosumab group and the alendronate group were 398.0 and 332.0 mU/dL, respectively. Serum BAP, t-PINP, 1,25(OH)_2_VitD, pentosidine, and homocysteine levels were also not significantly different between the groups. The median T-score at the LS was -1.3, and the mean T-scores at the FN and UD were -1.5 and -1.7, respectively. BMDs of the various sites were not significantly different between the two groups. Summarizing the above, no significant differences were found in demographic and baseline clinical characteristics between the two groups.

**Table 2:** Laboratory data and bone mineral density (BMD) at baseline.

	Total	Denosumab	Alendronate	*P*
	(N = 28)	(N = 14)	(N = 14)	
**Serum biochemical markers**				
** Albumin (g/dL)**	3.9 (2.8–4.3)	4.0 (2.9–4.2)	3.6 (2.3–4.3)	0.764
** eGFR (ml/min/1.73m**^**2**^**)**	56.0 (44.9–72.4)	61.1 (46.2–86.1)	49.0 (44.1–61.2)	0.085
** Corrected Ca (mg/dl)**	9.6 ± 0.5	9.6 ± 0.5	9.6 ± 0.5	0.795
** P (mg/dL)**	3.2 ± 0.6	3.3 ± 0.5	3.2± 0.6	0.716
** Uric Acid**	5.7 ± 1.3	5.6 ± 1.6	5.7 ± 1.1	0.760
** ALP (U/L)**	226.1 ± 69.6	219.0 ± 56.3	233.2 ± 82.4	0.598
** TG (mg/dL)**	169.4 ± 121.8	154.6 ± 75.6	184.1 ± 156.9	0.532
** TC (mg/dL)**	235.8 ± 117.5	222.3 ± 121.2	249.3 ± 116.7	0.552
** HDL-C (mg/dL)**	62.4 ± 16.4	59.9 ± 15.3	65.0 ± 17.6	0.423
** LDL-C (mg/dL)**	113.5 (86.8–138.3)	105.0 (81.3–126.3)	124.0 (100.2–170.8)	0.102
** LDL / HDL ratio**	1.8 (1.2–2.6)	1.8 (1.2–2.5)	2.0 (1.3–3.0)	0.629
** HbA1c**	5.9 ± 0.6	5.9 ± 0.7	5.9 ± 0.5	0.798
** Intact PTH (pg/mL)**	44.2 ± 25.0	41.5 ± 21.1	46.9 ± 28.9	0.580
** TRACP-5b (mU/dL)**	339.0 (248.0–543.5)	398.0 (248.0–528.5)	332.0 (204.5–591.3)	0.800
** Total P1NP (ug/L)**	36.4 (25.3–52.7)	43.0 (26.7–52.8)	30.4 (23.9–54.6)	0.783
** BAP (ug/L)**	10.4 (7.1–13.7)	10.5 (7.2–14.3)	10.4 (6.7–11.6)	0.645
**1,25(OH)**_**2**_ **VitD**	43.6 ± 21.1	39.1 ± 19.1	47.6 ± 22.8	0.295
** Pentosidine (ug/L)**	0.04 ± 0.02	0.04 ± 0.02	0.04 ± 0.02	0.872
** Homocysteine (nmol/mL)**	13.3 (11.3–19.0)	13.8 ± 5.0	24.8 ± 21.8	0.232
**Bone mineral density**				
** Lumbar spine**				
**Measurement—g/cm** ^**2**^	0.889 (0.787–1.022)	0.895 (0.745–1.060)	0.875 (0.821–1.045)	0.918
** T score**	-1.3 (-2.1–-0.2)	-1.3 (-2.5–0.3)	-1.2 (-1.9–-0.4)	0.918
** Femoral neck**				
**Measurement—g/cm** ^**2**^	0.676 (0.532–0.716)	0.672 ± 0.17	0.627 ± 0.11	0.715
** T score**	-1.5 ± 1.2	-1.3 ± 1.3	-1.7 ± 0.9	0.378
**Ultra-distal radius**				
**Measurement—g/cm** ^**2**^	0.400 ± 0.11	0.409 ± 0.12	0.390 ± 0.09	0.323
** T score**	-1.7 ± 1.5	-1.5 ± 1.6	-1.9 ± 1.5	0.473

Data are presented as mean ± SD, median (range from 25th to 75th percentile). Abbreviations: eGFR, estimated glemerular filtration rate; P, phosphate; ALP, alkaline phosphatase; TG, triglyceride; TC, total cholesterol; HDL-C, HDL-cholesterol; LDL-C, LDL-cholesterol; HbA1c, glycated hemoglobin; intact PTH, intact parathyroid hormone; TRACP-5b, tartrate-resistant acid phosphatase 5b; t-P1NP, total- type I collagen N-terminal propeptide; BAP, bone-specific alkaline phosphatase

### Effects of denosumab and alendronate on bone turnover makers

**Fig 2:**
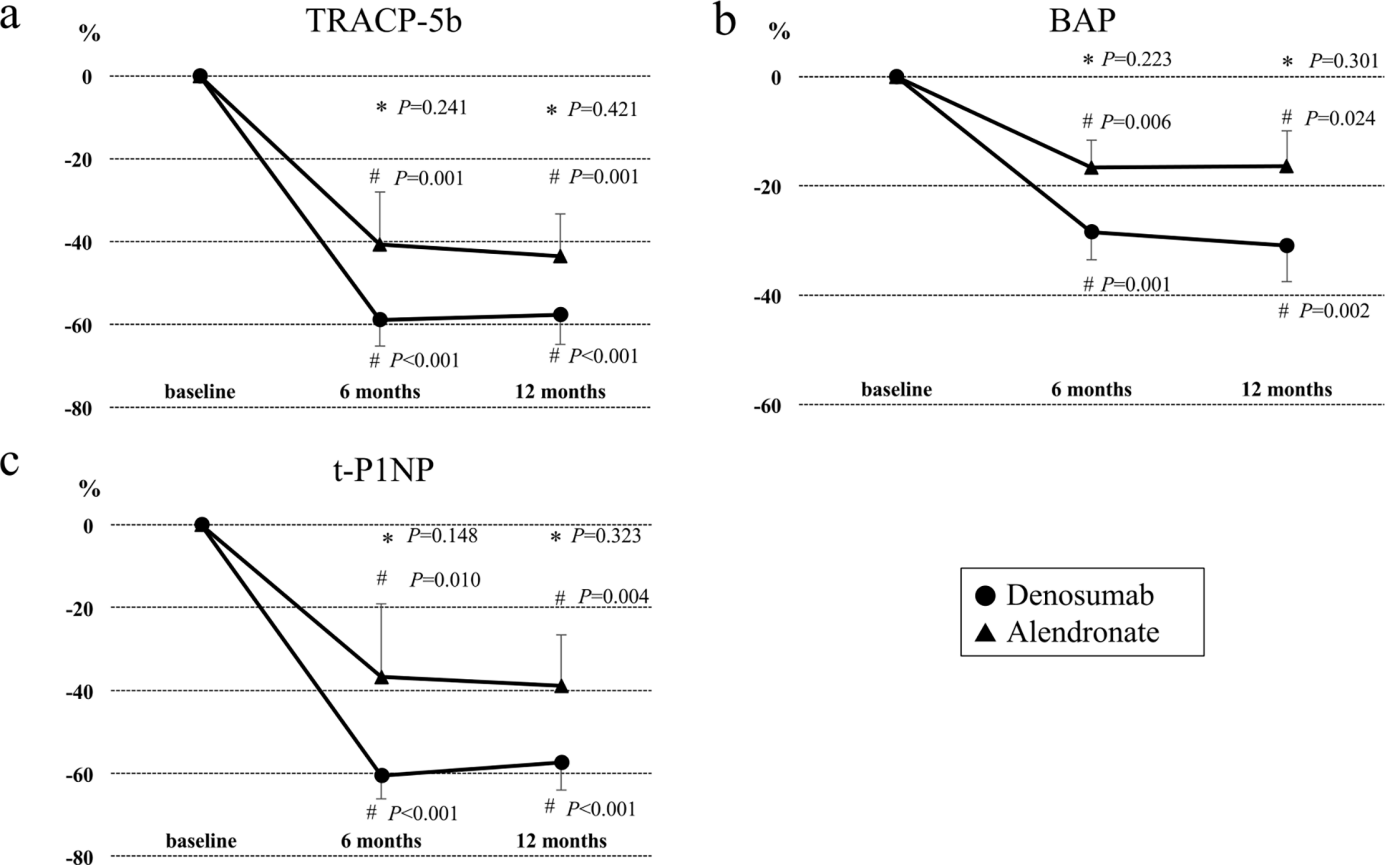
Effects of denosumab and alendronate on bone turnover makers. Percent changes (means ± SEM) in bone turnover markers from baseline to 12 months in the denosumab and alendronate groups. *P compared with the alendronate group (by Wilcoxon’s rank sum test). ^#^*P* compared with baseline (by Wilcoxon’s signed rank test). Abbreviations: TRACP-5b, tartrate-resistant acid phosphatase 5b; BAP, bone-specific alkaline phosphatase; t-PINP, total-type I collagen N-terminal propeptide.

**Fig 2** show the percentage changes (mean ± SEM) from baseline in bone turnover makers during the 12-month treatment period. After denosumab treatment, large decreases of serum TRACP-5b (-58.9%, p<0.001), BAP (-28.5%, p<0.01), and t-PINP (-60.6%, p<0.001) were found at 6 months compared to baseline, as well as at 12 months (TRACP-5b: -57.7%, p<0.001; BAP: -30.9%, p<0.01; t-PINP: -57.4%, p<0.001). In patients in the alendronate group, the levels of TRACP-5b (6 months: -40.6%, p<0.01; 12 months: -43.5%, p<0.01), BAP (6 months: -16.6%, p<0.01; 12 months: -16.3%, p<0.05), and t-PINP (6 months: -36.7%, p<0.05; 12 months: -38.9%, p<0.05) were significantly decreased during the study period. When the two groups were compared, denosumab treatment tended to decrease the bone turnover markers more than alendronate treatment, but the trend was not significant.

### Effects of denosumab and alendronate on BMD

**Fig 3:**
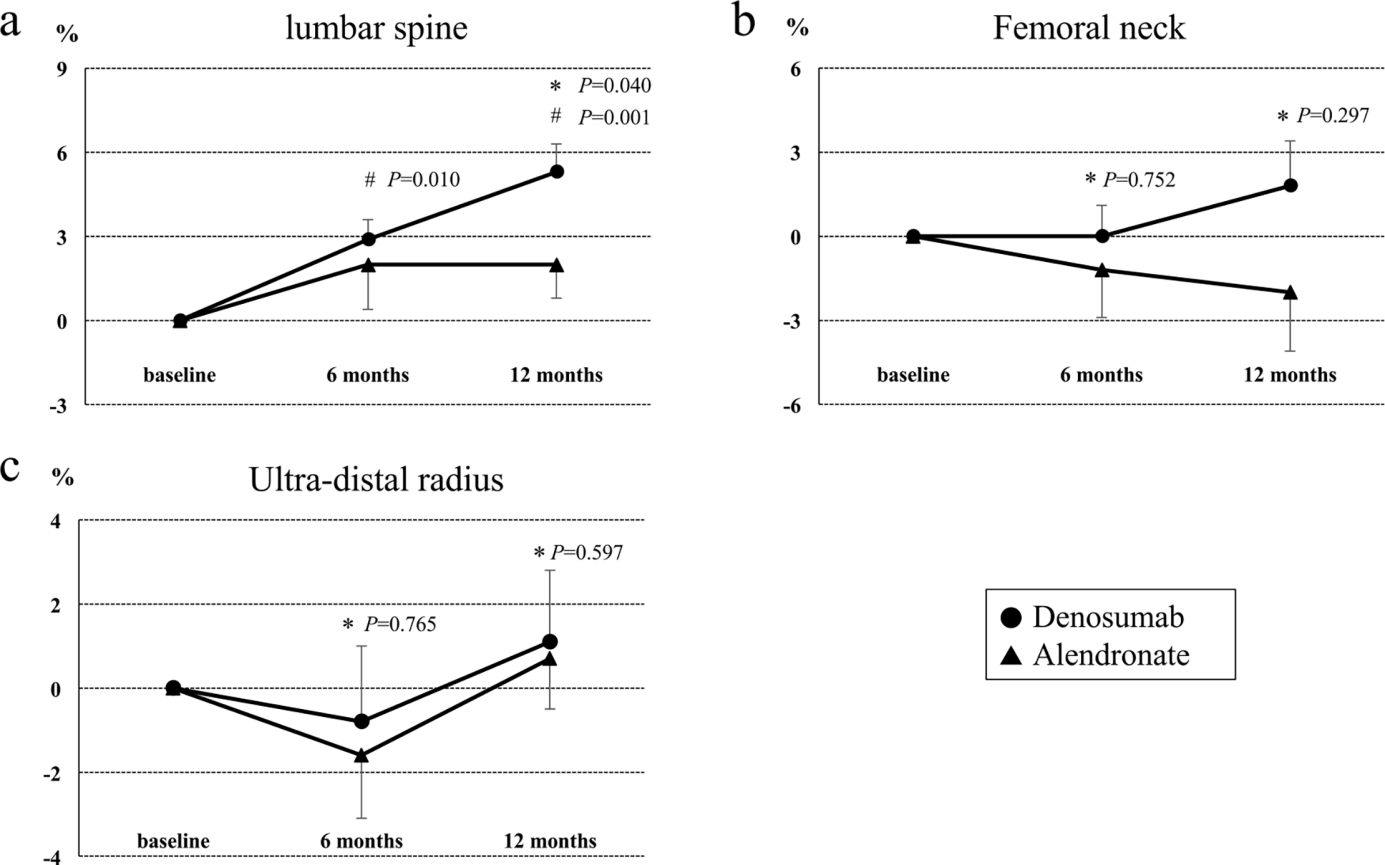
Effects of denosumab and alendronate on BMD. Percent changes (mean ± SEM) in bone mineral density from baseline to 12 months in the lumbar spine (A), femoral neck (B), and ultra-distal radius (C) in the denosumab and alendronate groups. *P compared with the alendronate group (by Wilcoxon’s rank sum test). ^#^*P* compared with baseline (by Wilcoxon’s signed rank test).

**Fig 3** show the percentage changes (mean ± SEM) from baseline in BMDs of the LS (L2-4), FN, and UD. In the LS, 6 and 12 months of treatment with denosumab significantly increased BMD compared to baseline (6 months: +2.9%±0.7%, p<0.05; 12 months: +5.3%±1.0%, p<0.01), whereas no significant difference was found at other sites (FN 6 months: +0%±1.1%, p = 0.750; FN 12 month: +1.8%±1.1%, p = 0.141; UD 6 months: -0.8%±1.8%, p = 0.289; UD 12 months: +1.1%±1.7%, p = 0.966). When compared to alendronate, a marked increase of LS BMD was found in the denosumab group at 12 months (p<0.05). On the other hand, alendronate treatment was not significantly different between baseline and post-treatment at the various sites during the study period (LS 6 months: +2.0%±1.6%, p = 0.424; LS 12 months: +2.0%±1.2%, p = 0.315; FN 6 months: -1.2%±1.7%, p = 0.748; FN 12 months: -2.0%±2.1%, p = 0.554; UD 6 months: -1.6%±1.5%, p = 0.265; UD 12 months: +0.7%±1.2%, p = 0.349).

### Adverse events

During the study period, two serious adverse events (SAEs; worse skin rash (n = 1), and pulmonary tuberculosis (TB; n = 1)) and two adverse events (hypocalcemia (n = 2)) were observed in the denosumab group, while there were no adverse events, including gastric distress, in the alendronate group. Asymptomatic hypocalcemia (Common Terminology Criteria for Adverse Events (CTCAE) grade 1) developed after administration of denosumab and was attenuated immediately by additional daily calcium and calcitriol. One patient having chronic eczema withdrew consent from this study, because the eczema deteriorated one month after starting denosumab. The eczema improved gradually without treatment. One patient who developed TB about ten months after first denosumab administration was treated with a combination of anti-TB drugs.

## Discussion

In the present study, the major findings were as follows. First, denosumab treatment significantly decreased the serum levels of bone turnover markers and increased LS BMD compared to baseline from 6 months, while BMDs at the FN and UD were not significantly different from baseline. In addition, after 12 months of denosumab treatment, a much greater increase of LS BMD was found than with alendronate treatment. Second, although bone turnover markers were markedly suppressed in patients treated with alendronate, no significant differences in BMD at various sites were observed between before and after treatment.

The pathogenesis of GIOP is complex and has not been clearly elucidated. It has been reported that the increase of Wnt antagonists induced by glucocorticoids suppressed osteoblast differentiation and maturation and promoted osteoblast apoptosis [12]. In contrast to the effects on osteoblasts, glucocorticoids promoted osteoclastogenesis and prolonged osteoclast survival through the suppression of osteoprotegerin (OPG) and enhancement of RANKL [13]. Reduced bone formation and increased apoptosis of osteocytes are the crucial difference between GIOP and postmenopausal osteoporosis, which is characterized by increased bone resorption. Although bisphosphonate is widely used as a key drug for GIOP and postmenopausal osteoporosis, it has been reported that the increase of BMD with bisphosphonate treatment was of a lower magnitude in GIOP patients than in postmenopausal osteoporosis patients [4].

Recently, a few studies have shown the therapeutic potential of denosumab for GIOP[12,13]. First, Dore et al. reported that denosumab increased LS and hip BMD and reduced serum type I C-telopeptide (sCTx-I) and PINP compared to placebo in patients with rheumatoid arthritis (RA) receiving glucocorticoids for 12 months. They reported that denosumab markedly increased BMD in RA patients receiving glucocorticoids as effectively as in non-glucocorticoid users with RA [14]. Moreover, the efficacy of switching from oral bisphosphonates to denosumab in chronic glucocorticoid users was reported by Mok et al. [15]. They showed that 12 months of denosumab treatment led to a large gain of spinal BMD compared to continuation of bisphosphonates, while minor infections were more common in the denosumab group. The most prescribed bisphosphonates were alendronate (79%), followed by risedronate (12%), in their study. The present study is the first randomized, controlled trial in which only alendronate was selected as a control drug and all patients had not been prescribed bisphosphonates before the study. These studies, including the present study, indicate that denosumab is superior to alendronate in increasing LS BMD for GIOP patients regardless of previous bisphosphonate use. In the present study, the bone turnover markers were likely more suppressed in the denosumab group, although that difference was not significant. A previous study showed that denosumab suppressed bone turnover markers (β-CTX and PINP) more strongly than bisphosphonates in patients with GIOP [15]. Bone turnover markers are considered to be a useful tool for monitoring the response to treatment. In fact, t-PINP was found to be the most meaningful marker for assessing later increases in LS BMD (data not shown), which is consistent with a previous report [16].

Previous studies have shown that alendronate increased LS and FN BMDs and reduced vertebral fractures in patients with GIOP [3,17,18]. This evidence supports the use of alendronate as a first-line drug for the treatment of GIOP. However, poor adherence to treatment is a weak point of oral alendronate therapy. Indeed, noncompliant patients receiving alendronate had a markedly higher risk of hip fracture than compliant patients over the 4-year observation period in the retrospective analysis of Taiwanese patients with osteoporotic vertebral fractures [19]. Recently, Freemantle et al. reported a 2-year, randomized, crossover study that compared adherence between subcutaneous denosumab (60 mg every 6 months) and oral alendronate (70 mg once weekly) in postmenopausal women. Patients preferred injections of denosumab to oral alendronate therapy because of the frequency of administration, the mode of administration, and convenience, and denosumab achieved significantly better adherence, compliance, and persistence than alendronate [20]. Since poor adherence to anti-osteoporosis drugs increases fracture risk, administration of denosumab could avoid this problem.

In this way, denosumab could become a first-line drug for GIOP. However, adverse events including infection with denosumab have not been fully understood. Indeed, serious adverse events of cellulitis were more frequently observed in the denosumab treatment group than in the placebo group in postmenopausal women with osteoporosis [6]. As for GIOP patients, a significantly higher number of upper respiratory infections was reported than with bisphosphonates [15]. Furthermore, Bonani et al. performed a one-year, randomized, controlled trial to evaluate the safety of denosumab in kidney transplant patients treated with immunosuppressive agents. They reported that urinary tract infections (UTIs), which were more commonly observed in the denosumab treatment group than in the control group, largely occurred in the first 6 months after kidney transplantation [21]. Although TB was observed in one patient treated with denosumab in the present study, TB was not noted in these reports. They speculated that a higher number of these minor infections might be induced by the inhibition of the RANK/RANKL pathway by denosumab. However, all of the study periods in these reports, including the present study, were short. Further studies to evaluate the long-term safety of denosumab in patients treated with immunosuppressive therapy should be undertaken.

In the present study, an FN fracture occurred in 1 patient treated with denosumab, but in none of those treated with alendronate during the follow-up period. The patient with an FN fracture had been given two courses of intravenous methylprednisolone pulse therapy for relapse of antineutrophil cytoplasmic antibody (ANCA)-associated vasculitis (AAV).

Although the present study provided important insights into the efficacy of denosumab for GIOP, several shortcomings should be discussed. First, since the study subjects were all Japanese GIOP patients with glomerular disease, the efficacy of denosumab may not be generalizable to other populations. Second, the size of the study population was small, and the primary outcome was the percent change in BMD, not the incidence of new fracture. Thus, the superiority of denosumab to alendronate in preventing new fractures including vertebral fractures in patients with GIOP could not be evaluated. Moreover, it was difficult to perform the stratified analysis by underlying glomerular disease subtype. Third, 25(OH)VitD, which is a barometer of vitamin D status, was not measured, because only 1,25(OH)_2_VitD measurement is approved in Japan. Fourth, other bone turnover markers whose levels are affected by the glomerular filtration rate were not evaluated because some patients in this study had renal insufficiency. Fifth, this was a single-center, open-label study. Sixth, the present study included men, pre- and post-menopausal women, and patients treated with glucocorticoids for various periods, but the intention was to investigate the efficacy of denosumab in a setting as close as possible to the clinical practice setting.

In conclusion, denosumab treatment achieved marked suppression of bone turnover, which resulted in a greater increase of LS BMD than alendronate treatment. These results suggest that denosumab is a useful option for the treatment of GIOP. However, further studies with a larger sample size and a long-term study period are needed to evaluate the reduction of fracture risk with denosumab treatment in GIOP patients.

## Supporting information

S1 File The CONSORT checklist. (DOC)

S2 File The study protocol in Japanese. (DOCX)

S3 File The study protocol in English. (DOCX)
