# Supplementary material for: The effects of denosumab and alendronate on glucocorticoid-induced osteoporosis in patients with glomerular disease: A randomized, controlled trial
Source: PLoS One. 2018 Mar 15;13(3):e0193846. doi: 10.1371/journal.pone.0193846 (PMC5854344; doi:10.1371/journal.pone.0193846)
Supplement: S2 File — (DOCX) [file pone.0193846.s002.docx]

**研究実施計画書**

**1 研究の名称**

腎炎患者におけるステロイド性骨粗鬆症に対する抗RANKL抗体製剤デノスマブとビスフォスフォネート製剤アレンドロネートの有効性と安全性のランダム割付による並行群間比較試験

**2 研究の実施体制（研究機関の名称及び研究者等の氏名を含む。）**

**2-1）研究者名（昭和大学内）**

研究責任者 医学部内科学講座腎臓内科学部門　　　　准教授　　　　伊與田　雅之

分担研究者 医学部内科学講座腎臓内科学部門　　　　教授　　　　　 柴田　孝則

医学部内科学講座腎臓内科学部門　　　　助教　　　　　　 松本　啓

医学部内科学講座腎臓内科学部門　　　　大学院生　　 井芹　健

＜研究事務局＞

昭和大学腎臓内科医局

伊與田 雅之（研究事務局代表）、柴田　孝則、松本　啓、井芹　健

〒111-1111東京都品川区旗の台1-5-8

電話：03-3784-8000（内線8533）、FAX：03-3784-5934

　E-mail：iyoda@med.showa-u.ac.jp（伊與田 雅之）

**2-2）個人情報管理責任者**

　　　　　　　医学部内科学講座腎臓内科学部門　　　　　　　　助教　　　眞田　大介

**3 研究の目的及び意義(当該研究の臨床的意義を明記)**

　ステロイド性骨粗鬆症を合併する腎炎患者を対象とし、ビスフォスフォネート製剤であるアレンドロネート35mgのweekly経口投与、あるいは抗RANKL抗体製剤デノスマブ60mgの6ヶ月間隔皮下投与を12ヶ月間実施した際の骨粗鬆症の改善効果をランダム割付による並行群間比較試験にて検討する。また、試験薬投与により低カルシウム血症の発生が危惧される為、両群とも試験期間中カルシトリオール0.25μgの１日１回の経口投与を行い、血清カルシウムの値により主治医が適宜投与量を調整する。

二重エネルギーX線吸収測定法（DEXA法）にて計測する骨密度の投与前からの変化量を主要評価項目として評価し、投与6、12ヶ月後の骨密度、骨代謝マーカー、骨折の有無、投与継続率、GFR低下率ならびに発現したすべての有害事象を副次評価項目として評価する。

**4 研究の方法及び期間**

**4-1）実施施設**

試験は昭和大学病院腎臓内科病棟および外来で実施し、腎炎患者の通常診療範囲内の採血、二重エネルギーX線吸収測定法(DEXA法)にて得られた検査結果を用いて行う。

**4-2）評価項目**

**1）主要評価項目（Primary endpoint）：**

二重エネルギーX線吸収測定法（DEXA法）にて計測する骨密度の投与前からの変化量

**2）副次評価項目（Secondary endpoint）**

　　骨代謝マーカー、骨折の有無、投与継続率、GFR低下率ならびに発現した全ての有害事象

**3）安全性評価項目**

　　低カルシウム血症及び発現した全ての有害事象

**4-3）試験薬概要（用法・用量、投与期間、予測される副作用）**

試験薬：デノスマブ製剤 （プラリア：［第一三共株式会社］）

用法：用量：60mgを6ヶ月間隔にて皮下投与

投与期間：12ヶ月

予測される副作用：低カルシウム血症7 例（0.8％）、背部痛7 例（0.8％）、γ-GTP上昇7 例（0.8％）、高血圧7 例（0.8％）、湿疹6 例（0.7％）、関節痛5 例（0.6％）等（添付文書より抜粋）

対照薬：アレンドロネート製剤 （ボナロン：［帝人ファーマ］）

用法：用量：35mgを1週間間隔にて経口投与

投与期間：12ヶ月

予測される副作用：上腹部痛4件(2.4％)、胃不快感4件(2.4％)、胃潰瘍3件(1.8％)、胃炎3件(1.8％) （添付文書より抜粋）

併用薬：カルシトリオール製剤　(ロカルトロール：［中外製薬］)

用法：用量：0.25μgを1日1回経口投与

投与期間：12ヶ月

予測される副作用：そう痒感19件(0.43%)､BUN上昇17件(0.39%)､血中クレアチニン上昇15件(0.34%)､AST(GOT)上昇14件(0.32%)､嘔気13件(0.30%)等（製品副作用頻度表より抜粋）

**4-4）試験薬の割付**

本試験は割付によって治療群間に医学的背景の差が生じぬようにランダム化比較試験にて実施する。

割付責任者はあらかじめ、http://www.randomization.com/ にてアレンドロネート（ボナロン）群とデノスマブ（プラリア）群を1：1の割合にて1ブロック2症例（1症例 vs 1症例）のブロック割付を行う。試験担当医師の症例組入れ速度に差異が生じる可能性と、外来診察時に同意取得がなされた場合に、速やかに割付を完了する必要があることから、割付は本試験を担当する試験担当医師毎に40症例分（10例分の予備を含む）作成し、個々の担当医師毎に配布された40症例分の割付コード表を適切に保管する。

作成した割付コード表に従い、各試験担当医師毎に「割付指示・確認書」（別紙1参照）をNo. 1～No. 40を作成しそれぞれを小封筒に封入、封筒表紙には当該コードを使用する試験担当医師名と割付番号を記載する。すなわち「伊No.1～伊No.40」「柴No.1～柴No.40」「その他No.1～他No.40」の3セットを作成する。

また、本試験は研究対象者の背景が割付け時の交絡因子として混入する影響を軽減するため、以下の項目を層別因子として設定する。

層別因子

・ 性別：男性、女性

- 年齢：65歳以上、65歳未満
- 閉経：閉経前、閉経後

小封筒2ごとに中封筒を20セット作成する。中封筒の表紙に想定される以下の層別因子のパターンを記載し、該当する症例の同意取得が得られた場合には該当するパターンの中封筒から順次小封筒を取り出し使用する。中封筒内の2つの小封筒が終了した場合、新たな中封筒（パターン記載のないもの）に追加の層別因子パターンを記載し、順次使用する。

想定される層別因子パターン

・「男性、65歳以上」「男性、65歳未満」

・「女性、65歳以上、閉経後」「女性、65歳以上、閉経前」

・「女性、65歳未満、閉経後」「女性、65歳未満、閉経前」

試験担当医師は同意取得後、順次小封筒を開封し封筒内の「割付指示・確認書」から当該被験者の試験薬の割付を確認し、速やかに「割付指示・確認書」の“実施日と実施者欄（自署）”を記載する。なお試験担当医師は、被験者から試験参加の同意取得し割付を行った場合、割付が順番通り適切に行われたことを担保するため、「割付指示・確認書」に記載した割付日（小封筒開封日）と同意取得に関する記録（同意した旨と同意日）ならびに被験者割付群を診療録に記載する。

「割付指示・確認書」は研究分担者（井芹）がすべての「割付指示・確認書」を適切に保管する。

割付事務局（割付責任者）

昭和大学臨床薬理研究所　龍 家圭

〒157-8577　世田谷区北烏山6-11-11　TEL: 03-3300-5247　FAX: 03-3300-1653

**4-5）試料（情報）と入手方法**

試験開始前

被験者の組み入れ時において、診療録から下記の情報を収集する。

被験者背景：年齢（生年月日）、性別、身長・体重、血圧、現病歴に関する情報（腎生検（病理）

所見、治療内容（投薬内容））

また、臨床検査ならびに骨量に関する下記のデータをあわせて収集する。

血液検査：血清Cr、eGFR、i-PTH、ALP、Ca、P、TRACEP5b、BAP、P1NP(Ⅰ型プロプロコラーゲン

－N－プロペプチド)、ペントシジン、ホモシステイン、1.25(OH)2ﾋﾞﾀﾐﾝD

骨塩量の測定部位（二重エネルギーX線吸収測定法：DEXA法）：1/3 radius、femoral neck、lumbar

spine

試験薬投与後

試験薬投与後、6、12ヶ月後

血液検査：血清Cr、eGFR、i-PTH、ALP、Ca、P、TRACEP5b、BAP、P1NP(Ⅰ型プロプロコラーゲン

－N－プロペプチド)、ペントシジン、ホモシステイン、1.25(OH)2ﾋﾞﾀﾐﾝD

試験薬投与後、6、12ヶ月後

骨塩量の測定部位（二重エネルギーX線吸収測定法：DEXA法）：1/3 radius、femoral neck、lumber

spine

なお、試験担当医師による被験者の安全性確認（バイタルサインの確認、有害事象の聴取）は通

常診療における経過観察に準じて試験期間中を通して行なう。

試験中のスケジュール

| 評価項目 | **試験**  **開始日** | **６ヶ月後** | **12ヶ月後** |
| --- | --- | --- | --- |
| 同意取得 | ● |  |  |
| 背景調査 | ● |  |  |
| 適格性確認 | ● |  |  |
| 血液・尿検査 | ● | ● | ● |
| 骨塩量測定  レントゲン検査 | ● | ● | ● |
| 副作用の調査 |  | | |

**4-6）試験中止基準**

　試験担当医師は、試験期間中に下記に該当する被験者が発生した場合には、当該被験者に対する試験を中止する。また試験の中止又は中断を決定した時は、被験者に対する適切な対応をするとともに、速やかに機関の長にその理由とともに文書で報告する。

1）被験者より中止の申し入れがあった場合

2）被験者の都合により試験が中断された場合（転居、転医・転院、多忙、追跡不能等）

3）試験開始後、被験者が対象症例ではないことが判明した場合

4）偶発的な事故が発生した場合

5）有害事象が発現し（原疾患の増悪、合併症の増悪又は偶発症を含む）、試験担当医師が中止すべきと判断した場合

6）効果不十分又は症状悪化のため、試験の継続が困難となった場合

7）本試験実施計画書から重大な逸脱があり評価不能と判断される場合

8）被験者が試験担当医師の指示どおり服薬していないことが判明した場合

9）その他、試験担当医師が試験の継続を困難と判断し中止が妥当と判断した場合

**4-7）解析方法**

結果の解析は、同意取得後に実施した試験治療開始前の各観察項目をコントロールとして、試験薬投与後**6、12**ヶ月後に施行した血液検査、DEXA法などからコントロールからの変化量について試験治療の群間比較を行う。本試験の評価項目として設定した種々の検査・観察項目のデータのカテゴリーに従い、適切な統計解析を、統計ソフトを用いて解析比較する。

設定の際に考慮する。

**4-8）研究期間**

医学部における人を対象とする研究等に関する倫理委員会承認後、病院長の実施許可を得てから2018年3月31日まで

**5 研究対象者の選定方針**

**5-1）選択基準**

1. 昭和大学病院腎臓内科に入院または通院中の腎炎患者で、ステロイド性骨粗鬆症診断基準を満たす患者。
2. 同意取得時の年齢が20歳以上である患者。

**5-2）除外基準**

１）デノスマブ投与禁忌な患者。

２）アレンドロネート投与禁忌な患者。

３）悪性腫瘍を併発する患者。

４）半年以内にビスフォスフォネート製剤を投与した事がある患者。

５）半年以内にデノスマブを投与した事がある患者。

６）試験薬投与前の検査において、eGFRが35 mL/分/1.73m^2^以下の患者。

７）試験薬投与前の検査において、i-PTH 300 pg/mL以上の患者。

　８) 試験薬投与前の検査において、補正カルシウム値8.4 mg/dL以下の患者。

９）試験担当医師の判断により不適格と判断した者。

**5-3）目標症例数**

目標症例数は約30名程度

（デノスマブ投与群：15名、アレンドロネート投与群：15名）。

**5-4）設定根拠**

　本試験の担当医師の外来において、適格被験者の推定総数は現時点で約40症例である。保険適応内で実施する試験である一方で、ランダム割付を行うことから治療選択ができないことを被験者候補が懸念する場合も想定される。これらの状況ならびに今後に適格症例が新規患者として受診する可能性などを勘案して、実施可能最大症例数として30例を設定した。

また、ステロイド長期使用患者におけるデノスマブとビスフォスフォネート製剤の並行群間試験(Mok CC et al,Bone.2015;75:222–228)では約40症例と小規模で実施されていることも設定根拠の一つとして考慮に加えた。

**6 研究の科学的合理性の根拠**

**対象疾患についての説明**：

　腎炎は、多量の蛋白尿や血尿等を引き起こし、全身浮腫や腎機能悪化など多彩な臨床症状を呈する。腎炎に対して副腎皮質ステロイド薬や免疫抑制剤などの治療が中心となっているが、高容量、長期間副腎皮質ステロイド薬を使用することも稀ではなく、むしろ一般的であり副腎皮質ステロイド薬の副作用が問題となる。ステロイド性骨粗鬆症はADL低下につながる非常に重要な副作用の１つであり、長期ステロイド治療を受けている患者の30-50%に骨折が起こるとの報告(ステロイド性骨粗鬆症ガイドライン)もある。

**対象疾患の従来の治療法とその問題点**：

　アレンドロネートは、代表的な骨粗鬆症治療薬であるが、上部消化管問題(胃部不快感など)、長期服薬のアドヒアランス不良(起床時内服が必要であり、拒薬が多い)、高度腎機能障害には使用禁忌などの問題がある。

試験薬についての説明：2013年に新規骨粗鬆症薬として抗RANKL抗体製剤デノスマブが承認を受け、６ヶ月に一度の投与であり治療継続率の向上や腎機能低下例でも使用可能などの有用性が報告されている。デノスマブは、原発性骨粗鬆症にてスタンダードな薬剤であるビスフォスフォネート製剤を上回る効果が報告されている。

**本試験を計画するに至った経緯と、本試験で解決しようとする問題点**：

　2013年に新規骨粗鬆症薬としてデノスマブが発売となり、原発性骨粗鬆症においてビスフォスフォネート製剤(Nakamura T et al,J Clin Endocrinol Metab. 2014;7:2599–2607)はもとり、そのほかPTH製剤(Leder BZ et al, Lancet. 2015;2:S0140-6736(15)61120-5)などよりも骨密度改善効果、椎体骨折抑制効果が報告されている。また、今年に入り、長期ステロイド使用患者のビスフォスフォネート製剤からのデノスマブへの切り替えを行った所、骨密度の優位な改善効果を認め、安全面も問題なく有用であったとの報告も出てきている。(Mok CC et al,Bone.2015;75:222–228) デノスマブは、上記のように原発性骨粗鬆症に対してはエビデンスがあるもののステロイド性骨粗鬆症や腎炎患者におけるエビデンスはまだまだ不足しており、デノスマブの有用性、安全性を検証する為に本研究を施行する。

**7 インフォームド・コンセントを受ける手続等（インフォームド・コンセントを受ける場合には、同規定による説明及び同意に関する事項を含む。）**

1）事前に昭和大学医学部人を対象とする研究等に関する倫理委員会で承認の得られた説明文書･同意文書を研究対象者に渡し、文書及び口頭による十分な説明を行い、研究対象者の自由意思による同意を文書で得る。

2）研究対象者の同意に影響を及ぼすと考えられる有効性や安全性等の情報が得られた時や、研究対象者の同意に影響を及ぼすような実施計画等の変更が行われる時は、速やかに研究対象者に情報提供し、試験等に参加するか否かについて研究対象者の意思を予め確認するとともに、事前に人を対象とする研究等に関する倫理委員会の承認を得て説明文書・同意文書等の改訂を行い、研究対象者の再同意を得る。

3）説明文書・同意文書には、研究対象者が理解しやすい表現に配慮し作成する。

**8 個人情報等の取扱い（匿名化する場合にはその方法を含む。）**

本試験は、ヘルシンキ宣言に基づく倫理的原則及び人を対象とする医学系研究に関する倫理指針（2014年12月22日厚生労働省）に従い、本試験実施計画書を遵守して実施する。

本研究で取り扱う試料・情報等は、研究責任者が連結可能匿名化したうえで、研究・解析に使用する。匿名化の方法については、試料・情報から個人を識別できる情報（氏名、住所、生年月日、電話番号など）を削除し独自の符号を付す作業を行う。個人と符号の対応表は個人情報管理責任者が保管する。

**9 研究対象者に生じる負担並びに予測されるリスク及び利益、これらの総合的評価並びに当該負担及びリスクを最小化する対策**

本研究への参加の際、使用する薬物による副作用発現が被験者への危険性として想定される。起こりうる副作用については、研究実施計画書の「4 研究の方法及び期間」の4-3)試験薬概要」を参照。その他、本研究では試験薬の割付をランダムに行うことから、投与薬がデノスマブになった場合には、対照薬（ボナロン）と比較して年間で約5954円の負担増が想定される。しかしながら、これまでの報告によるとアレンドロネートを使用した場合には、デノスマブと比較して消化器症状が出現することが多いため、それらの治療に要する医療費を考えると両薬物間で医療費の負担差は限定的と考える。

**10 試料・情報（研究に用いられる情報に係る資料を含む。）の保管及び廃棄の方法**

**10-1）試料の保管及び廃棄の方法**

本研究終了後において、本研究で得られた被験者試料を他の研究において使用することはない。研究終了時には、全ての試料は速やかに破棄する。

**10-2）情報の保管及び破棄の方法**

被験者の本研究終了後に継続する通常医療活動において活用される従来の診療情報については、医師法等の関連法規に従い保管する。本研究の実施のために匿名化され取得した研究関連情報については、研究責任者あるいは分担研究者の所属する施設のコンピューターを用いて移動媒体内に保存するか、外部から切り離されたコンピューターのハードディスク内に保存する。情報を取り扱う研究者は、研究情報を取り扱うコンピューター及び移動媒体をパスワード管理するなどにより、情報の紛失・漏洩等に十分配慮した取扱いの上での保管を行う。

**10-3）情報の保管期間**

研究責任者は、試験終了後、速やかに医療機関の長に試験の終了報告書を提出するとともに、研究等の実施に係わる重要な文書（申請書類の控え、病院長からの通知文書、各種申請書・報告書の控、同意書、症例報告書、その他データの信頼性を保証するのに必要な書類又は記録等）を、研究の中止又は終了後少なくとも5年間、あるいは研究結果発表後3年が経過した日までの間のどちらか遅い期日まで保存する。

なお、通常診療に用いる医療情報の保管・破棄は関連法規（医師法）等の規定に従うこととする。

**11 研究機関の長への報告内容及び方法**

試験薬との因果関係の有無にかかわらず、重篤な有害事象が発現した場合は、試験担当医師は、安全確保を第一優先に迅速かつ適切な処置を講じた後、速やかに昭和大学病院 病院長及び昭和大学医学部人を対象とする研究等に関する倫理委員会委員長に報告するとともに、病院長による厚生労働大臣への報告ならびに公表について協力する。

また、研究の実施状況について１年に１回以上「研究終了/経過報告書(研究)」を用いて研究機関の長に報告する。

**12 研究の資金源等、研究機関の研究に係る利益相反及び個人の収益等、研究者等の研究に係る利益相反に関する状況**

本試験の計画、実施、発表に関して可能性のある利益相反（conflict of interest）はない。利益相反とは、研究成果に影響するような利害関係を指し、金銭及び個人の関係を含む。

本研究は、昭和大学医学部内科学講座腎臓内科学部門が計画し実施する自主臨床研究であり本研究に使用する医薬品の製造販売を行っている製薬会社をはじめ、他の団体からの資金的援助に基づいて行われるものではない。

**13 研究に関する情報公開の方法**

**13-1）研究実施計画書の登録**

本臨床研究は、ヘルシンキ宣言ならびに人を対象とする医学系研究に関する倫理指針（2014年12月22日厚生労働省）を遵守して実施することから、当該臨床研究の内容を公表するため、国立大学附属病院長会議(UMIN-CTR）が設置している公開データベースに、臨床研究計画の登録を行うこととする。

**13-2）知的財産権及び研究結果の公表について**

この研究から特許権、また、それを基として経済的利益が生じる可能性があるが、その権利は研究を実施する研究機関や研究者に属し、本試験の被験者がこの権利を持つことはない。また本試験実施計画書に基づいて行われた試験成績は、研究会及び実施医療機関の共有のものとする。成績の公表に関する事項は、研究会により決定する。公表の際には被験者の個人情報を保全する。

**14 研究対象者等及びその関係者からの相談等への対応**

本研究に同意した後でも、疑問や不明な点があった場合は、自由に研究者への質問を受け入れる。研究者がその時点で得られる情報をもとに返答する。また、本研究の計画及び方法についての資料はいつでも閲覧可能とする。

≪連絡先≫

担当者：井芹　健（昭和大学内科学講座腎臓内科学部門

　　　　　　03（3784）8533（平日8:30～17:00，土曜8:30～13:00）

　　　　　　03（3784）8000（休日・時間外）：腎臓内科当直医

**15 代諾者等からインフォームド・コンセントを受ける場合**

本研究では成人を対象とし、また被験者本人による文書同意の取得を必須とすることから、代諾者による同意は該当しない。

**16 インフォームド・アセントを得る場合**

成人かつ理解能力がある人を対象としているので、アセント取得は該当しない。

**17緊急かつ明白な生命の危機が生じている状況における研究の実施（指針第12の5の規定）**

生命の危機が生じている状況での研究は想定しておらず、該当しない。

**18 研究対象者等に経済的負担又は謝礼がある場合には、その旨及びその内容**

本研究実施に伴う医療費は保険診療で行なうが、一部の研究費用は講座研究費を用いる。

すなわち本試験にともない実施される通常診療保険外の特殊検査である骨代謝マーカーの費

用については、被験者への費用負担とならないよう、試験開始前に事前に病院医事課と調整を

行う。特殊検査以外の通常診療行われている診察・検査・試験薬の処方等は、通常の保険診療

に準じて支払うものとし、被験者に負担がかからないように配慮して行う。

**19 侵襲（軽微な侵襲を除く。）を伴う研究の場合には、重篤な有害事象が発生した際の対応**

発生した有害事象の治療は、原則として通常の保険診療にて行うものとするが、治療におい本試験の実施により被験者に有害事象（健康被害）が発生した場合には、試験担当医師は、十分な治療その他の適切な措置を行うと同時にその原因の究明に努め、カルテならびに症例報告票に齟齬なく記載する。また、試験薬の投与を中止した場合や、有害事象に対する治療が必要となった場合には、研究対象者にその旨を伝える。

発生した有害事象の治療は、原則として通常の保険診療にて行うものとする。

なお、試験薬との因果関係の有無にかかわらず、重篤な有害事象が発現した場合は、試験担当医師は、安全確保を第一優先に迅速かつ適切な処置を講じた後、速やかに昭和大学病院長および昭和大学医学部人を対象とする研究等に関する倫理委員会委員長に報告するとともに、病院長による厚生労働大臣への報告ならびに公表について協力する。

**20 侵襲を伴う研究の場合には、当該研究によって生じた健康被害に対する補償の有無及びその内容**

本試験の実施により研究協力者に健康被害が生じた場合には、研究責任者又は分担研究者は十分な治療等の適切な措置を行うとともに、その原因を究明に努める。医療費などの金銭的補償は行わない。また、本試験の実施にともない、医薬品副作用被害救済制度など規制当局への対応が必要となる健康被害が生じた場合、試験担当医師はこれに対処し、必要に応じて十分な協議を関係者と行うものとする。

**21 通常の診療を超える医療行為を伴う研究の場合には、研究対象者への研究実施後における医療の提供に関する対応**

試験終了後における治療制限はない。試験担当医師は、当該被験者の試験期間終了後の治

療効果を十分に勘案し、最善の治療の継続を行なうための治療選択を行なうこととする。

**22 研究の実施に伴い、研究対象者の健康、子孫に受け継がれ得る遺伝的特徴等に関する重要な知見が得られる可能性がある場合には、研究対象者に係る研究結果（偶発的所見を含む。）の取扱い**

　本研究において実施した検査・観察項目により、被験者の医療上の問題が偶発的に発見さ

れる場合が想定される。その場合には、被験者に対してその旨を十分に説明するとともに、

必要に応じて偶発的に発見された医療上の問題点に関する専門医への相談・紹介等を行う。

**23 研究に関する業務の一部を委託する場合には、当該業務内容及び委託先の監督方法**

本研究において、業務の一部を委託する事はしない為、該当しない。

**24 研究対象者から取得された試料・情報について、研究対象者等から同意を受ける時点では特定されない将来の研究のために用いられる可能性又は他の研究機関に提供する可能性がある場合には、その旨と同意を受ける時点において想定される内容**

　本研究終了後、本研究により取得した試料・情報等を、将来において新たに計画された研究への使用は想定していないため、本項目は該当しない。

**25モニタリング及び監査を実施（指針 第20の規定）する場合には、その実施体制及び実施手順**

　本研究におけるデータの品質管理・品質保証のためのモニタリング等は、個人情報管理責任者が以下の項目を主として、適切に取得・保管されていることを確認する。取得や保管に問題があることが確認された場合においては、速やかに研究責任者に報告し、改善措置を協議するとともに、必要に応じて医療機関の長ならびに人を対象とする研究等に関する倫理委員会の委員長に報告する。

取得・保管の確認事項

・被験者の同意文書の取得・保管状況

・被験者の選択基準・除外基準の適切性（評価項目と評価時期）

別添１

割付指示・確認書

症例 柴No.1は

割付：アレンドロネート群

にて 試験を実施する

上記の割付にて試験を実施しました

割付実施日：　　　年　　　月　　　日

実施者

（自署）

（実施者の自署署名の後、分担研究者：井芹に提出をお願いします）
