# Supplementary material for: The effects of denosumab and alendronate on glucocorticoid-induced osteoporosis in patients with glomerular disease: A randomized, controlled trial
Source: PLoS One. 2018 Mar 15;13(3):e0193846. doi: 10.1371/journal.pone.0193846 (PMC5854344; doi:10.1371/journal.pone.0193846)
Supplement: S3 File — (DOCX) [file pone.0193846.s003.docx]

**Study Protocol**

1. **Study title**

Effect of Denosumab and Alendronate on glucocorticoid-induced osteoporosis in patients with glomerulonephritis: a randomized controlled trial

1. **Research Center and Investigator**

**2-1）Principal Investigator**

Division of Nephrology, Department of Medicine, Showa University School of Medicine

Chief-Investigator 　 Associate professor　　　　Masayuki Iyoda

Investigator 　 　　Professor　　　 　 Takanori Shibata

　　 　Assistant Professor　　　 Kei Matsumoto

　 　　Clinical Fellow　　 Ken Iseri

＜Public contact＞

Division of Nephrology, Department of Medicine, Showa University School of Medicine

1-5-8 Hatanodai, Shinagawa-ku, Tokyo 142-8666, Japan

Tel: +81-3-3784-8533, Fax: +81-3-3784-5934

E-mail：iyoda@med.showa-u.ac.jp（Dr Masayuki Iyoda）

**2-2）Chief privacy officer**

　　　　　　　 　Assistant Professor　　　 Daisuke Sanada

**3. Background**

　　Glucocorticoids are widely used for the treatment of various inflammatory and autoimmune diseases, but they have various adverse effects on multiple organ systems. In addition, the risk of fractures is increased in patients treated with glucocorticoids. Glucocorticoid-induced osteoporosis (GIOP), characterized by rapid bone loss within the first months after initiation and slower bone resorption thereafter, is the most common form of secondary osteoporosis. It has been reported that the relative risk of fractures was much higher in patients receiving glucocorticoids independent of bone mineral density (BMD) than in those never treated with glucocorticoids, and long-term glucocorticoid therapy caused bone fractures in 30-50% of patients (1). As for bone protection, bisphosphonates are considered standard therapy for GIOP, but concerns with the use of bisphosphonates in children, young men, and premenopausal women, who are often included in the population with glomerulonephritis, still remain (2).

　　Denosumab is a fully humanized monoclonal IgG2 antibody against the receptor activator of nuclear factor-κB ligand (RANKL), which is a key effector of osteoclast formation, function, and survival. To date, denosumab has been widely used not only in postmenopausal osteoporosis, but also other types of osteoporosis, such as secondary hyperparathyroidism, but limited information is available regarding the therapeutic potential of denosumab in patients with GIOP. The purpose of the present study was to investigate the efficacy and safety of denosumab compared to alendronate on GIOP in patients with glomerular disease.

1. **Objectives**

　　The aim of this study is to evaluate the effect of denosumab and alendronate on glucocorticoidiunduced osteoporosis in patients with glomerulonephritis. This was a 12-month, single-center, open-label, randomized, controlled study. Subjects with glomerular disease who were diagnosed with GIOP according to Japanese Society for Bone and Mineral Research criteria were randomly assigned in a 1:1 ratio to receive denosumab (60 mg) subcutaneously every 6 months or oral alendronate 35 mg/week for 1 year. All patients received at least calcitriol 0.25 μg/day throughout the study. Medications for the treatment of the underlying diseases were continued as usual.

The primary outcome was percent change of bone mineral density (BMD) at 6, 12 months after intervention. Secondary outcomes were percent change of bone metabolism markers, rate of continuation, side effect at 6, 12 months after intervention.

**5. Methods**

**4-1）Medical institution**

This study was conducted in the Showa University Hospital.

**4-2）Endpoint**

**1）Primary endpoint**

Percent change of bone mineral density (BMD) at 6, 12 months after intervention

**2）Secondary endpoint**

　　Percent change of bone metabolism markers, rate of continuation, side effect at 6, 12

months after intervention

**3）Safety assesment**

　　All adverse events or reaction

**4-3）Study drugs**

Drug1：denosumab (60 mg) subcutaneously every 6 months.

(Daiichi-Sankyo, Tokyo, Japan)

Drug2：oral alendronate 35 mg/week for 1 year.

(Teijin, Tokyo, Japan)

**4-4）Randomization**

　　This study was conducted as randomized controlled trial so that patient’s background equal between treatment groups. Prior to the study, allocation officer who is independent of the study creates random allocation cards (total 40 cards) in a 1:1 ratio, the so-called block size is two, with stratification using Randomization.com (<http://www.randomization.com/)>. The stratification was done by sex, age and menopausal. He put random allocation cards into each envelope with serial numbers

and the card in the envelope is not visible from the outside of the envelope. Predetermined random list was kept in an inaccessible third place. When a subject is ready to be enrolled in the study, denosumab and alendronate will be randomly assigned according to a random allocation card. After allocation, date, patient ID and results after the procedure will be recorded by investigator.

Stratification factors

・ Sex：male、female

- Age：over 65yrs、under 65yrs
- menopausal：premenopausal、postmenopausal

<Allocation Officer>

Assistant Professor　　　 Kakei Ryu

Clinical Pharmacology, Pharmacology, Showa University School of Medicine

6-11-11 Kitakarasuyama, Setagaya-ku, Tokyo 157-8577, Japan

Tel: +81-3-3300-5247, Fax: +81-3-3300-1653

**4-5）Measurements**

**Patient characteristics**

Date of birth, gender, height, weight, blood pressure, complication, past medical history,

glomerular disease and drug therapy are obtained from medical record.

**Blood examinations:**

　Cr, eGFR, i-PTH, ALP, Ca, P, tartrate-resistant acid phosphatase 5b (TRACP-5b), bone-specific

　ALP (BAP), and total-type I collagen N-terminal propeptide (t-PINP), pentosidine、homocysteine、

　1.25(OH)_2_VitD

**Dual-energy X-ray absorptiometry (DXA; Discovery A):**

BMD of the lumbar spine(LS), femoral neck (FN), and ultra-distal radius (UD) are measured.

　Radiographs of the lateral lumbar and thoracic spine were also taken.

All subjects were questioned concerning adverse events, and serum chemistry and hematology values were evaluated at each visit.

**Study schedule**

| **Assessments** | **Baseline** | **6 months** | **12 months** |
| --- | --- | --- | --- |
| **Informed consent** | ● |  |  |
| **Patient’s background** | ● |  |  |
| **Confirmation of eligibility** | ● |  |  |
| **Laboratory test** | ● | ● | ● |
| **DXA**  **X-p** | ● | ● | ● |
| **Adverse events** |  | | |

**4-6）Termination criteria**

1）The subject offered to withdraw from the study.

2）Investigator judged that the study could not be continued because of subject’s convenience (for example; relocation, missed-follow up).

3) The subject who turned out did not meet the inclusion criteria.

4）The unexpected accident was occurred.

5）The serious adverse event developed and the investigator judged that the study should be discontinued.

6）The study could not be continued because of aggravation of underlying diseases.

7）Serious deviation from the study.

8）The subject who turned out could not comply with the prescribed administration of study drug.

9）Investigator judged that the study should be discontinued.

**4-7）Assessments**

The percentage change in BMD and bone turnover markers from baseline at 6 or 12 months are assessed. Data are analyzed by JMP Pro Ver.13 (SAS Institute, Cary, NC). Comparisons between two groups for normally distributed variables were performed using Student’s *t*-test, and the Wilcoxon rank sum test was used for non-normally distributed variables. Between-group differences in the mean change in BMD and bone turnover markers from baseline to 6 months or 12 months were examined by the Wilcoxon rank sum test. Similarly, within-group changes were assessed by paired *t-*tests. For all tests, the level of significance was set at p<0.05. All statistical analyses were performed by JMP Pro Ver.13 (SAS Institute, Cary, NC).

All adverse events were assessed, regardless of the determinations of causality by the investigators.

**4-8）Study period**

From the approval of the Showa University Ethics Committee to 31/03/2018

**5. Eligibility Criteria**

**5-1）Inclusion criteria:**

1) patients who meet the glucocorticoid- induced osteoporosis criteria

2) Patient whose age is more than 20 years old

**5-2）Exclusion criteria:**

1) Patients who are considered to be contraindicated for denosumab.

2) Patients who are considered to be contraindicated for alendronate.

3) Cancer patients.

4) Patients who have pretreated with bisphosphonate in recent 6 months.

5) Patients who have pretreated with denosumab in recent 6 months.

6) Patients whose eGFR is less than 35ml/min/1.73m2.

7) Patients whose i-PTH is more than 300ml/min.

8) Patients whose corrected Ca is less than 8.4 mg/dL.

9) Patients who are thought to be inappropriate for this study by physician.。

**5-3）Target sample size**

Target sample is 30 subjects.

（denosumab：15、alendronate：15）

**5-4）Sample size estimates**

　　In our hospital, the number of outpatients who meet eligibility criteria are about forty and this study is exploratory study. Furthermore, previous study with the small sample size (about 40 patietns were completed) regarding denosumab having superiority to alendronate in patients with chronic glucocorticoid users was reported (3). On the basis of these facts, the sample size in this study was determined.

1. **Human rights and ethical standards**

　　The study is conducted in accordance with ethical standards for human experimentation established by the Declaration of Helsinki Principles and with the ethics guild line in Japan. And also the study protocol and the informed consent form are approved by the Showa University ethics committee. This study was registered with the University Hospital Medical Information Network Clinical Trials Registry (UMIN-CTR) (number UMIN 000027182). All patients provided their written informed consent to the study following a careful explanation.

**7. References**

1. Weinstein RS. Glucocorticoid-Induced Bone Disease. N Engl J Med. 2011;365:62–70.

2. Rizzoli R, Biver E. Glucocorticoid-induced osteoporosis : who to treat with what agent ? Nat Publ Gr . 2014;11(2):98–109.

3. Mok CC, Ho LY, Ma KM. Switching of oral bisphosphonates to denosumab in chronic glucocorticoid users: A 12-month randomized controlled trial. Bone. 2015;75:222–8.
